# The Shear Bond Strength Between Calcium Silicate-Based Biomaterials and Glass Ionomer Restorative Materials: An In Vitro Comparative Study

**DOI:** 10.3390/jfb17060309

**Published:** 2026-06-22

**Authors:** Mehmet Salık, Elif Pınar Bakır

**Affiliations:** Department of Restorative Dentistry, Faculty of Dentistry, Dicle University, Diyarbakır 21280, Türkiye; elifpinarbakir@gmail.com

**Keywords:** calcium silicate-based biomaterials, glass ionomer cement, shear bond strength, interface morphology, in vitro study

## Abstract

**Aim:** The aim of this study was to comparatively evaluate the shear bond strengths between different calcium silicate-based biomaterials and glass ionomer-based restorative materials. **Materials and Methods:** In this in vitro study, a total of 96 acrylic blocks were prepared, each containing a standardized cylindrical cavity measuring 4 mm in diameter and 2 mm in depth. Four different calcium silicate-based biomaterials (ProRoot MTA, Biodentine, TheraCal LC, and MTA BioRep) were placed into the cavities according to the manufacturers’ instructions. Three different glass ionomer restorative materials (Fuji II LC, Equia Forte HT, and Riva Self Cure) were then applied onto the biomaterial surfaces using molds measuring 2 mm in diameter and 2 mm in height, resulting in 12 experimental groups (n = 8). After storage at 37 °C for 24 h, the shear bond strengths were measured using a universal testing machine. The data were analyzed using the Kruskal–Wallis and Mann–Whitney U tests with Bonferroni correction (*p* < 0.05). **Results:** The highest bond strength was observed in the TheraCal LC–Fuji II LC combination, whereas the lowest value was obtained in the MTA BioRep–Equia Forte HT group. Both the type of biomaterial and type of glass ionomer cement had a statistically significant effect on the bond strength (*p* < 0.05). **Conclusions:** The combination of calcium silicate-based biomaterial and glass ionomer-based restorative material influenced the early shear bond strength. These findings suggest that material selection may play an important role in early bonding behavior at the biomaterial–restorative material interface.

## 1. Introduction

Vital pulp therapy (VPT) encompasses minimally invasive approaches aimed at preserving pulp vitality and promoting the biological healing of the dentin–pulp complex affected by deep caries, trauma, or operative procedures [1,2,3]. The success of these treatments depends not only on clinical factors, such as accurate diagnosis and effective hemostasis, but also on the biological and mechanical properties of the pulp-capping materials used [4]. In this context, calcium silicate-based biomaterials are widely used in vital pulp therapy because of their biocompatibility, bioactive properties, and potential to promote mineralized tissue formation [5,6].

Nevertheless, achieving sufficient mechanical stability at the biomaterial–restorative material interface is important for reducing bacterial microleakage and preserving the early integrity of the restorative complex [7,8]. Glass ionomer-based restorative materials can be used as an intermediate layer or restorative material over calcium silicate-based pulp-capping materials because of their chemical adhesion to dental hard tissues, fluoride release, biocompatibility, and coefficient of thermal expansion similar to that of tooth structure [9,10]. However, conventional, high-viscosity glass hybrid and resin-modified glass ionomer systems may exhibit different bonding behaviors with calcium silicate-based biomaterials owing to their different setting mechanisms and surface characteristics.

The available literature on the bonding of calcium silicate-based materials to restorative materials has largely focused on resin composite systems, while data regarding their interaction with glass ionomer-based restorative materials remain limited [8,11,12]. Moreover, there is still insufficient evidence regarding the bonding performance of newer calcium silicate-based materials, such as MTA BioRep, and contemporary glass ionomer-based systems, such as Equia Forte HT. This underscores the need for a more detailed evaluation of the early interfacial behavior between these material groups, which may be used together in clinical practice.

ProRoot MTA and Biodentine are widely used clinically and have been extensively evaluated in experimental studies among hydraulic calcium silicate-based cements [6,13,14]. In contrast, TheraCal LC has a different setting mechanism and chemical composition because of its resin-modified and light-curable nature [15]. MTA BioRep is a newer calcium silicate-based biomaterial whose physicochemical and biological properties have been evaluated in a limited number of studies [16,17]. Comparing these different material groups with various glass ionomer-based restorative materials may contribute to a better understanding of the early bonding performance of different material combinations.

The application of adhesive systems may introduce an additional variable affecting the surface properties of calcium silicate-based materials, particularly during the early setting period. Therefore, evaluating the direct bonding behavior at the biomaterial–glass ionomer interface by eliminating the potential effect of an additional adhesive layer is important.

This study aimed to comparatively evaluate the shear bond strengths between different calcium silicate-based biomaterials and various glass ionomer-based restorative materials. The null hypothesis tested was that the type of biomaterial and type of glass ionomer-based restorative material would have no statistically significant effect on the shear bond strength in the absence of an additional adhesive system.

## 2. Materials and Methods

**Figure 1:**
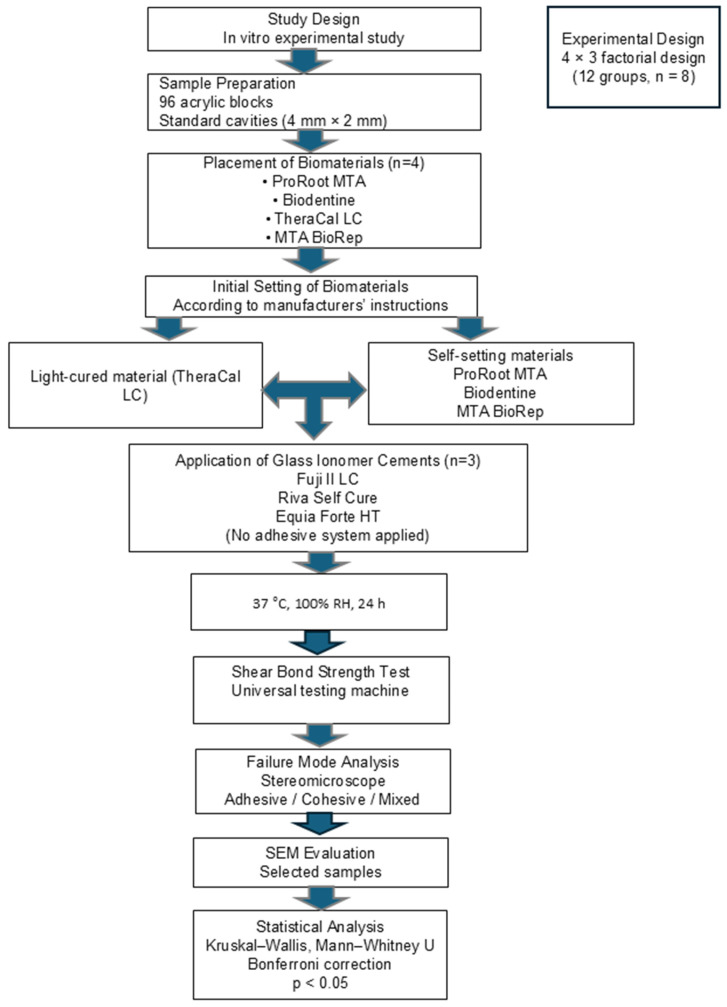
A flowchart showing the experimental design of this study and the main procedural steps from specimen preparation to statistical analysis.

In this in vitro study, the shear bond strengths between different calcium silicate-based biomaterials and glass ionomer-based restorative materials were comparatively evaluated. The experimental procedures were carried out at the Research Laboratory of Dicle University and the Laboratory Application and Research Center of Batman University. The experimental design and main procedural steps of this study are presented in Figure 1.

Four different calcium silicate-based biomaterials (ProRoot MTA, Biodentine, TheraCal LC, and MTA BioRep) and three different glass ionomer-based restorative materials (Fuji II LC, Equia Forte HT, and Riva Self Cure) were used in this study (Table 1). The experimental groups were formed by pairing each biomaterial with each glass ionomer-based restorative material, resulting in a total of 12 experimental groups.

The sample size was determined before this study using an a priori power analysis performed with G*Power software version 3.1.9.7. The power analysis was conducted based on a one-way ANOVA model for the overall comparison among the 12 experimental groups. The effect size was calculated from the mean and standard deviation values reported in a methodologically similar in vitro shear bond strength study [18]. Based on this reference study, Cohen’s f was estimated to be approximately 0.65. With α = 0.05, statistical power = 0.95, and 12 experimental groups, the minimum required total sample size was calculated as 72. Accordingly, eight specimens were used for each experimental group, and a total of 96 specimens were included in this study.

**Figure 2:**
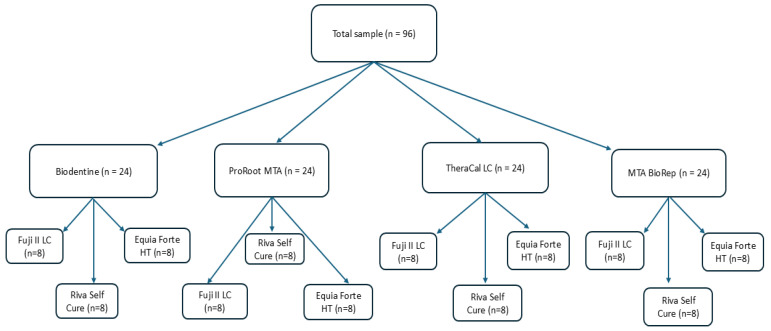
The distribution of the experimental groups according to biomaterial and glass ionomer-based restorative material combinations. A total of 96 specimens were divided into four biomaterial groups (n = 24); each biomaterial group was further divided into three glass ionomer-based restorative material subgroups (n = 8).

After numbering, the specimens were allocated to the experimental groups using a computer-generated random number sequence. A total of 96 self-curing acrylic resin blocks were used for the specimen preparation. In each block, a cylindrical cavity measuring 4 mm in diameter and 2 mm in depth was prepared, taking into account standardized specimen preparation protocols used in previous similar in vitro shear bond strength studies [8]. The calcium silicate-based biomaterials were prepared according to the manufacturers’ instructions and placed into these cavities (Figure 2).

To reduce operator-related variability, all specimen preparation, biomaterial placement, and glass ionomer-based restorative material application procedures were performed by the same researcher. For the TheraCal LC specimens, the material was applied in 1 mm thick increments, and each layer was light-cured for 20 s using an LED curing unit (Guilin Woodpecker Medical Instrument Co., Ltd., Guilin, Guangxi, China; light intensity: 1100 mW/cm^2^), positioned perpendicular to the material surface at an approximate distance of 1 mm. The ProRoot MTA, Biodentine, and MTA BioRep materials were prepared according to the manufacturers’ recommendations. A moist cotton pellet was placed over the material surface, and the specimens were sealed with a temporary filling material (Cavit; 3M ESPE, St. Paul, MN, USA). The ProRoot MTA, Biodentine, and MTA BioRep specimens were then stored at 37 °C under 100% humidity in an incubator (Mikrotest MST 55, Mikrotest Instrument, Ankara, Türkiye) for 4 h, 12 min, and 15 min, respectively, according to the manufacturers’ recommendations.

Subsequently, the corresponding glass ionomer-based restorative materials were applied onto the biomaterial surfaces using cylindrical molds measuring 2 mm in diameter and 2 mm in height, according to the manufacturers’ instructions. This mold size was selected by considering the standardized restorative material dimensions and bonding area approach used in previous in vitro shear/microshear bond strength studies [19,20].

Fuji II LC, a resin-modified glass ionomer cement, was light-cured for 20 s using an LED curing unit (Woodpecker, China; light intensity: 1100 mW/cm^2^), positioned perpendicular to the material surface at an approximate distance of 1 mm. The Equia Forte HT and Riva Self Cure specimens were allowed to set according to the manufacturers’ instructions to complete their setting reactions.

In this study, no adhesive system was used in order to eliminate the potential confounding effect of an additional adhesive layer at the biomaterial–restorative material interface and to evaluate the intrinsic bonding behavior between the materials.

**Figure 3:**
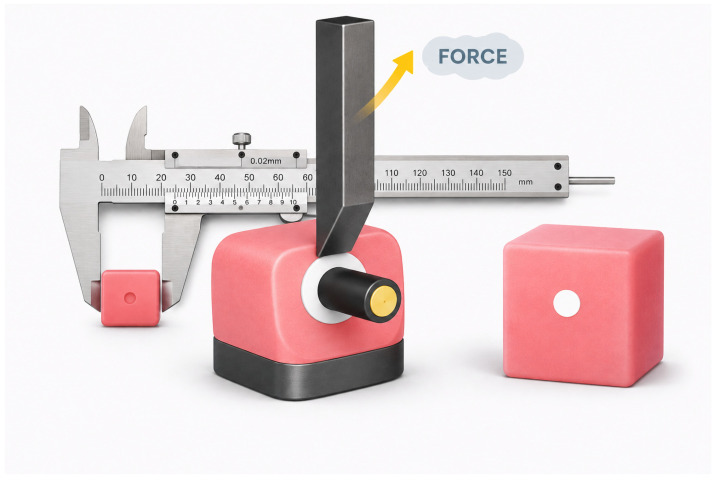
A schematic representation of the specimens prepared for the shear bond strength test and the testing setup. A calcium silicate-based biomaterial was placed into a standardized acrylic block, a glass ionomer-based restorative material cylinder was applied onto its surface, and loading was applied parallel to the interface using a chisel-shaped blade.

After application of the restorative materials, all specimens were incubated at 37 °C under 100% relative humidity for 24 h, in accordance with similar protocols reported in the literature [8]. Shear bond strength testing was performed using a universal testing machine (Shimadzu, Kyoto, Japan). Before testing, the specimens were fixed to the testing platform, and a chisel-shaped blade was positioned at the base of the restorative material cylinder, as close as possible to the biomaterial–restorative material interface. Loading was applied parallel to the interface at a crosshead speed of 1 mm/min until failure occurred (Figure 3). The shear bond strength was calculated in MPa by dividing the maximum force at failure (N), recorded by the testing machine, by the bonded surface area (πr^2^; r = 1 mm).

After testing, the fractured surfaces were examined under a stereomicroscope (Carl Zeiss, Oberkochen, Germany) by a single observer who was blinded to the group allocation. The failure modes were classified as adhesive failure, cohesive failure within the biomaterial, cohesive failure within the glass ionomer-based restorative material, and mixed failure. To assess the intra-observer reproducibility, failure mode classifications were repeated by the same observer after a two-week interval without access to the initial evaluation results. Agreement between the first and second evaluations was assessed using Cohen’s kappa coefficient.

In addition, to evaluate the interfacial morphology, one specimen from each experimental group was selected using simple random sampling (total n = 12). The selection was performed using a random number table after the specimens had been numbered. The selected specimens were analyzed by scanning electron microscopy (SEM) (FEI Company, Hillsboro, OR, USA).

Statistical analyses were performed using IBM SPSS Statistics version 31.0 (IBM Corp., Armonk, NY, USA). The normality of the data was assessed using the Shapiro–Wilk test, and the homogeneity of variance was evaluated using Levene’s test. Since the data were not normally distributed, non-parametric tests were used. Overall group comparisons were performed using the Kruskal–Wallis test. When a statistically significant difference was detected with the Kruskal–Wallis test, pairwise comparisons were performed using the Mann–Whitney U test. To reduce the risk of type I error in multiple comparisons, Bonferroni correction was applied, and significance was evaluated based on Bonferroni-adjusted *p* values. For the overall analyses, the level of statistical significance was set at *p* < 0.05. To assess the magnitude of between-group differences, eta-squared (η^2^) and epsilon-squared (ε^2^) effect size values were calculated based on the H statistic obtained from the Kruskal–Wallis test. The intra-observer agreement for the failure mode classifications was analyzed using Cohen’s kappa coefficient.

## 3. Results

Descriptive statistics of the measured shear bond strength values for all experimental groups are presented in Table 2. The lowest mean value was observed in the MTA BioRep–Equia Forte HT group (0.37 ± 0.21 MPa), whereas the highest mean value was obtained in the TheraCal LC–Fuji II LC group (16.41 ± 2.42 MPa).

**Figure 4:**
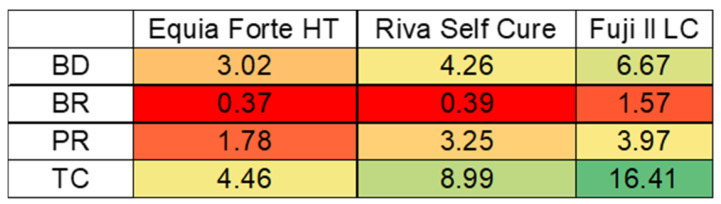
Heatmap showing mean shear bond strength values according to biomaterial–glass ionomer restorative material combinations. Values within cells represent mean MPa values, and color scale indicates transition from lower to higher bond strength values. BD: Biodentine; PR: ProRoot MTA; TC: TheraCal LC; BR: MTA BioRep.

**Figure 5:**
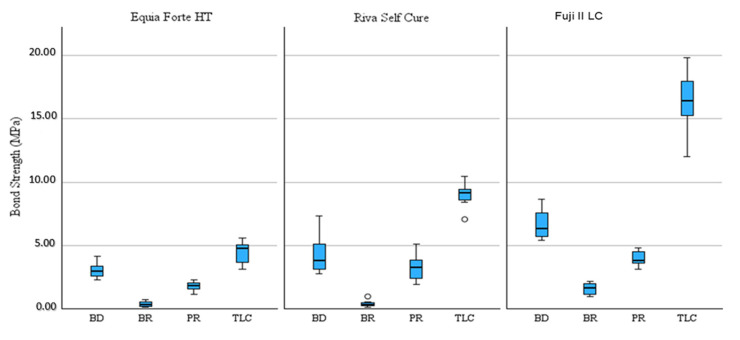
Boxplot showing median and distribution of shear bond strength values according to biomaterial type within each glass ionomer-based restorative material group. Open circles indicate outlier values. BD: Biodentine; BR: MTA BioRep; PR: ProRoot MTA; TLC: TheraCal LC.

**Figure 6:**
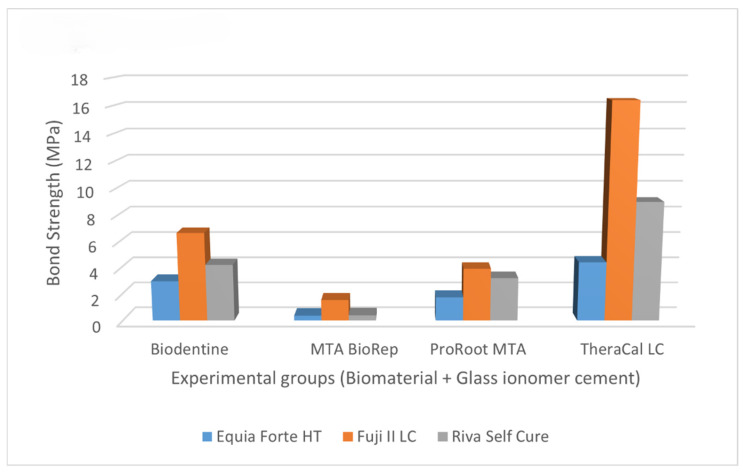
Bar chart showing mean shear bond strength values according to biomaterial–glass ionomer-based restorative material combinations. Bars represent mean MPa values.

The shear bond strength values obtained are presented as a heatmap in Figure 4, as a boxplot in Figure 5, and as a bar chart in Figure 6 to visually illustrate the differences among the groups.

In the overall comparison among the 12 experimental groups, a statistically significant difference was found in the measured shear bond strength values (Kruskal–Wallis test, H = 88.117; df = 11; *p* < 0.001). Effect size analyses showed that the experimental group variable had a very large effect on the shear bond strength (η^2^ = 0.949; ε^2^ = 0.942). Bonferroni-adjusted Mann–Whitney U post hoc analyses identified statistically significant pairwise differences among the experimental groups (Table 3).

Subgroup analyses revealed significant differences according to both the biomaterial type and glass ionomer-based restorative material type (Kruskal–Wallis test, *p* < 0.001 for all analyses). The pairwise comparison results among the biomaterials when the glass ionomer-based restorative material type was kept constant are presented in Table 4, while the pairwise comparison results among the glass ionomer-based restorative materials when the biomaterial type was kept constant are presented in Table 5.

**Figure 7:**
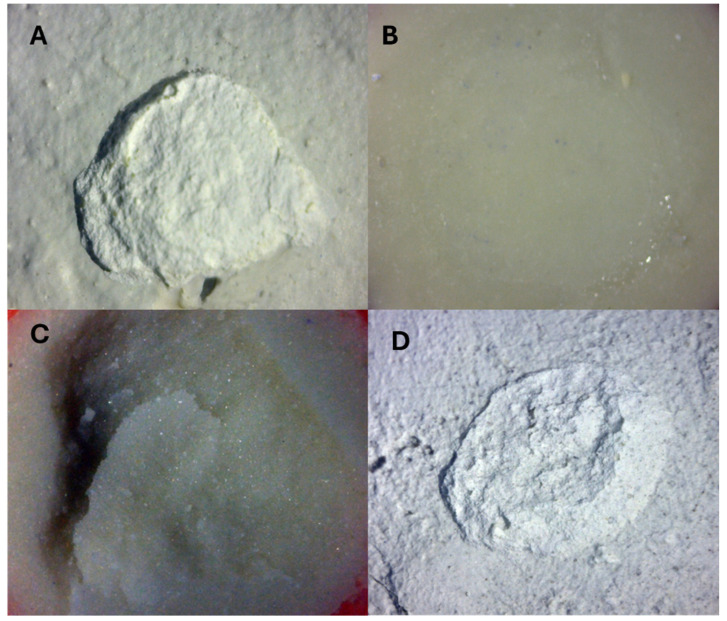
Representative stereomicroscopic images of failure modes observed in different calcium silicate-based biomaterial–glass ionomer combinations: (**A**) ProRoot MTA–Riva Self Cure; (**B**) TheraCal LC–Equia Forte HT; (**C**) TheraCal LC–Fuji II LC; (**D**) MTA BioRep–Fuji II LC.

**Figure 8:**
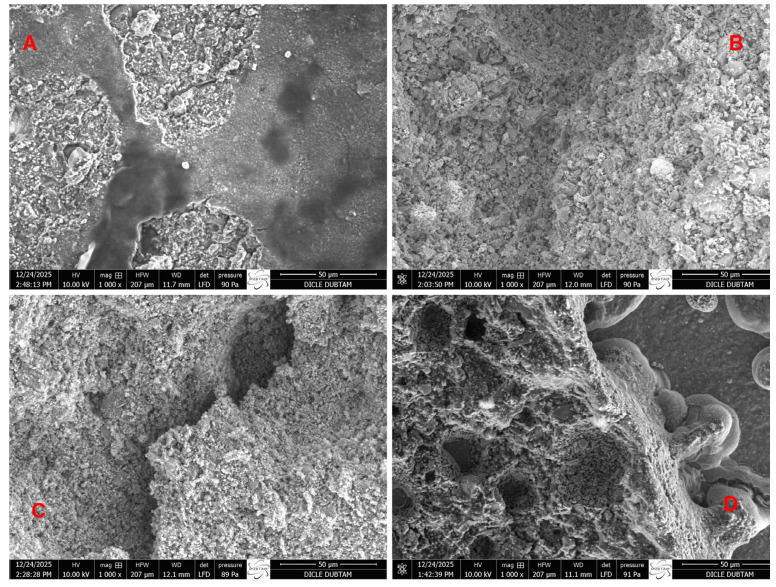
Representative SEM images showing interfacial morphology and failure patterns of different biomaterial–glass ionomer cement combinations (1000×): (**A**) TheraCal LC–Fuji II LC; (**B**) MTA BioRep–Equia Forte HT; (**C**) ProRoot MTA–Fuji II LC; (**D**) Biodentine–Riva Self Cure.

The failure mode distributions varied according to the biomaterial–glass ionomer-based restorative material combinations (Table 6). The intra-observer agreement for the failure mode classifications was very high (Cohen’s κ = 0.966; n = 96). In the ProRoot MTA groups, failures were predominantly cohesive within the biomaterial, while cohesive failures were also frequently observed in the MTA BioRep groups. However, in the MTA BioRep–Equia Forte HT group, adhesive failures and cohesive failures within the biomaterial were equally distributed. In the TheraCal LC groups, the failure mode varied according to the glass ionomer-based restorative material used; in the TheraCal LC–Equia Forte HT group, adhesive failure occurred in all specimens. In the Biodentine groups, adhesive, cohesive, and mixed failure modes were observed in varying proportions. Stereomicroscopic and SEM images of selected representative specimens are presented in Figure 7 and Figure 8.

## 4. Discussion

In this study, the early shear bond strengths between different calcium silicate-based biomaterials and glass ionomer-based restorative materials were comparatively evaluated without the application of an additional adhesive system. The findings showed that the shear bond strengths varied significantly according to both the biomaterial type and glass ionomer-based restorative material type. Therefore, the null hypothesis of this study was rejected.

In the literature, a substantial proportion of studies investigating the bonding behavior between calcium silicate-based materials and restorative materials have focused on resin composite systems and adhesive protocols; however, studies evaluating their direct interaction with glass ionomer-based restorative materials remain limited [8,11,12]. Although previous studies have examined ProRoot MTA, Biodentine, Medcem MTA, NeoMTA, and similar materials, to the best of our knowledge, no study has directly evaluated the bonding performance of MTA BioRep to glass ionomer-based restorative materials [8,13,14,21]. Therefore, the comparative evaluation of MTA BioRep with ProRoot MTA, Biodentine, and TheraCal LC may contribute to a better understanding of the early bonding performance of this material with glass ionomer-based restorative materials.

In the present study, the highest shear bond strength was obtained in the TheraCal LC–Fuji II LC combination. This finding may be related to the resin-modified and light-curable nature of TheraCal LC, which differs from that of conventional hydraulic calcium silicate-based cements [15]. This methacrylate-based structure may have contributed to the formation of a more compatible interface with Fuji II LC, a resin-modified glass ionomer cement, and to increased chemical/micromechanical interactions [22,23]. Accordingly, when comparing the bonding performance of TheraCal LC with that of hydraulic cements such as ProRoot MTA and Biodentine, its different setting mechanism and resin content should be taken into consideration.

In contrast, the lower bond strength observed in the TheraCal LC–Riva Self Cure combination compared with Fuji II LC may be related to the resin-free conventional glass ionomer structure of Riva Self Cure, which may have limited its interaction with the methacrylate-based phase of TheraCal LC. This finding is consistent with previous studies reporting that resin-modified glass ionomer cements may exhibit higher bond strength than conventional glass ionomer cements [11]. In glass hybrid systems, the bonding behavior may vary depending on the material formulation. Umale et al. reported a higher bond strength for the combination of TheraCal LC with standard Equia Forte [22]. However, the use of Equia Forte HT in the present study limits direct comparison of the results. This suggests that differences in the composition, viscosity, and setting characteristics of glass ionomer systems may influence bonding behavior.

In the Biodentine groups, the highest bond strength was obtained with Fuji II LC, whereas Riva Self Cure showed intermediate values and Equia Forte HT showed lower bond strength values. During the early hydration process of Biodentine, the formation of calcium hydroxide and the subsequent development of apatite-like calcium phosphate phases may create a surface with potential for ionic and chemical interactions with restorative materials [24]. The resin-modified matrix of Fuji II LC may have contributed to the formation of a more compatible interface with this calcium-rich surface. In contrast, in glass ionomer systems without a resin phase, bonding depends mainly on acid–base reactions and ionic interactions, which may have resulted in more limited bond strength values at the early stage. This finding is consistent with previous studies reporting that resin-modified glass ionomer cements may exhibit higher bond strengths than conventional glass ionomer cements [13,25].

The ProRoot MTA–Fuji II LC combination showed higher bond strength values than the ProRoot MTA combinations with Riva Self Cure and Equia Forte HT. However, the values obtained in the ProRoot MTA groups were generally lower than those in the TheraCal LC–Fuji II LC and Biodentine–Fuji II LC groups. These findings are consistent with studies reporting that the bond strength between ProRoot MTA and glass ionomer–based materials may be limited [14,26]. The relatively higher values obtained in the ProRoot MTA–Fuji II LC group may be explained by the additional micromechanical interaction potential provided by the resin phase of resin-modified glass ionomer cements. In contrast, resin-free or high-viscosity glass ionomer systems have been reported to show more limited interfacial adaptation to the MTA surface [8]. In addition, these materials may absorb water from the MTA surface during the early period, thereby affecting the hydration process and weakening interfacial integrity [18,27]. This mechanism may explain the lower bond strength values observed for ProRoot MTA, particularly in combination with Equia Forte HT.

Among the biomaterials evaluated in this study, MTA BioRep showed the lowest bond strength values across all glass ionomer-based restorative material combinations. This finding indicates that the early surface reactivity and interfacial formation capacity of MTA BioRep may differ from those of the other calcium silicate-based materials.

Although the modified formulation of MTA BioRep was developed to provide ease of use and a shorter setting time, literature data regarding the effects of these properties on its bonding behavior with restorative materials remain limited [16]. Previous microscopic analyses have reported that this material shows limited interaction with the dentin surface and that distinct tag-like structures, observed in some bioceramic materials, are not evident [17]. This suggests that the early surface characteristics of MTA BioRep may have limited its ability to establish sufficient mechanical adaptation and ionic interaction with the glass ionomer-based restorative materials. Indeed, although resin-modified glass ionomer cements have generally been reported to have higher bonding potential, this advantage was limited in the MTA BioRep–Fuji II LC combination in the present study [28]. Similarly, the low values obtained with Equia Forte HT and Riva Self Cure may also indicate weak early interfacial interaction between the MTA BioRep surface and glass ionomer-based restorative materials [29]. Taken together, these findings suggest that the reduced bonding performance of MTA BioRep may be associated with its formulation, setting kinetics, and early surface characteristics.

When the biomaterial type was kept constant, significant differences in the bond strengths were observed among the glass ionomer-based restorative materials. Overall, Fuji II LC showed higher bond strength values than Riva Self Cure and Equia Forte HT. This may be related to the ability of resin-modified glass ionomer cements to provide additional micromechanical interactions through their resin phase, in addition to the acid–base reaction [30,31]. Conversely, in resin-free glass ionomer cements, bonding depends mainly on acid–base reactions and ionic interactions, which may have resulted in more limited interfacial interaction with the biomaterial surface at the early stage. The lower values observed in the Equia Forte HT groups may be explained by the possible effects of the composition, filler ratio, and flow characteristics of glass hybrid systems on surface adaptation [32,33].

SEM and stereomicroscopic evaluations showed that the failure patterns varied according to the material combinations. In the TheraCal LC–Equia Forte HT group, adhesive failures characterized by separation along the interface were observed, whereas in the ProRoot MTA–Fuji II LC and MTA BioRep–Fuji II LC groups, cohesive failure patterns progressing mainly within the material were noted. In the Biodentine–Riva Self Cure and TheraCal LC–Fuji II LC groups, mixed failure morphologies involving both interfacial and intramaterial fracture extensions were observed.

Failure mode analysis is used as a complementary assessment to aid the interpretation of mechanical data obtained from bond strength tests [34,35]. However, failure mode distributions should be interpreted cautiously together with shear bond strength values. The presence of cohesive failures does not always indicate stronger interfacial bonding; particularly when observed together with low bond strength values, the measured value may also be influenced by the intrinsic cohesive strength of the material. Therefore, the shear bond strength values obtained in the present study reflect not only true interfacial adhesion but also the measured mechanical behavior of the biomaterial–restorative material complex under the test conditions.

This point is particularly important for the MTA BioRep groups. In these groups, the observation of cohesive failures together with low bond strength values indicates that failure may have been influenced not only by the interface but also by the internal structure of the material itself. Therefore, failure mode analysis should be considered as a supportive assessment of the bond strength results and should not be interpreted alone as direct evidence of interfacial bonding.

In this study, the shear bond strength was evaluated 24 h after application of the restorative material. This time point was selected to assess the early bonding behavior at the biomaterial–glass ionomer-based restorative material interface and to allow comparability with similar in vitro studies [8,22]. However, because the present study evaluated only the 24 h early bond strength, the findings may not directly reflect long-term bonding performance. In addition, delayed restoration has been reported to alter the bonding behavior of some calcium silicate-based materials [18]. Therefore, further studies including different restoration timings, longer waiting periods, and aging protocols are needed.

The use of standardized acrylic blocks instead of dentin allowed the cavity dimensions, bonding area, and specimen geometry to be kept consistent across all experimental groups. This approach enabled the biomaterial–glass ionomer-based restorative material interface to be evaluated under more controlled conditions. Nevertheless, since the tubular structure, moisture content, and organic–inorganic composition of dentin may influence bonding behavior, the results obtained using acrylic blocks may not fully reflect clinical dentin conditions [36]. Indeed, it has been reported that an interfacial layer may form when calcium silicate-based materials come into contact with dentin, that the thickness of this layer may vary depending on environmental conditions, and that elemental changes may occur at the material–dentin interface [37]. Therefore, if natural dentin had been used, the surface adaptation, interfacial interaction, and measured bond strength values of the materials might have differed. Future studies using natural dentin substrates will contribute to a better evaluation of the clinical relevance of the present findings.

No adhesive system was used in the present study in order to evaluate the direct bonding behavior at the biomaterial–glass ionomer-based restorative material interface. Since conventional and resin-modified glass ionomer-based restorative materials may be applied as an intermediate layer or restorative material over calcium silicate-based biomaterials in vital pulp therapy, the present adhesive-free experimental protocol may be relevant to certain clinical application scenarios [9,10,38,39]. However, the use of an adhesive layer may introduce additional variables, such as the type of adhesive system, etching protocol, application technique, solvent content, and layer thickness. These variables may affect the surface properties of calcium silicate-based biomaterials, particularly during the early stage of ongoing surface maturation, as well as their interfacial interaction with glass ionomer-based restorative materials. Therefore, the present study aimed to compare the intrinsic bonding performance between the materials while reducing the potential confounding effects of adhesive systems. Similarly, some in vitro studies have directly evaluated the bond strength between calcium silicate-based biomaterials and glass ionomer-based restorative materials without applying a separate adhesive system [21,22]. Moreover, because adhesive systems may also be used in clinical practice, the present findings should not be directly extrapolated to adhesive restorative protocols. Indeed, adhesive application has been reported to influence the bond strength between calcium silicate-based biomaterials and glass ionomer cements [8]. Therefore, further studies comparing adhesive and adhesive-free protocols are warranted.

By nature of the in vitro conditions, these findings should not be directly generalized to clinical success. Nevertheless, our results indicate that the bonding behavior between calcium silicate-based biomaterials and glass ionomer-based restorative materials may vary depending on the material combination. Accordingly, the selection of both the pulp-capping material and the overlying restorative material may be an important factor to consider for early interfacial stability in vital pulp therapy.

## 5. Limitations

This study has several limitations. First, the in vitro design does not fully reflect the complex conditions of the clinical environment. Thermal changes, occlusal loading, pulpal pressure, moisture control, and biological interactions were not simulated in this study. In addition, the preparation of specimens on standardized acrylic blocks rather than natural dentin limited the evaluation of the effects of dentin heterogeneity, tubular structure, and moisture content on the bonding behavior.

Only the 24 h early shear bond strength was evaluated in this study, and no long-term aging protocols were applied. Because the present study evaluated only a single early time point, the results may not fully reflect long-term bonding behavior. Furthermore, relying solely on the shear bond strength test may not be sufficient to fully characterize the mechanical behavior of the biomaterial–restorative material interface.

No adhesive system was used in this study in order to evaluate the direct/intrinsic bonding behavior between the materials. However, because adhesive protocols may also be preferred in clinical practice, the findings should not be directly generalized to adhesive restorative approaches. Future studies using natural dentin substrates and including different restoration timings, adhesive and adhesive-free protocols, alternative mechanical testing methods, and long-term aging conditions are needed.

## 6. Conclusions

The type of calcium silicate-based biomaterial and type of glass ionomer-based restorative material significantly affected the shear bond strength at the biomaterial–restorative material interface. Among the combinations tested, the highest bond strength was obtained in the TheraCal LC–Fuji II LC group, whereas the MTA BioRep groups generally exhibited lower bond strength values.

The findings indicate that the interaction between the surface characteristics of the biomaterial and the setting mechanism of the restorative material may play an important role in early interfacial bonding behavior. Therefore, in vital pulp therapy, the combined evaluation of the pulp-capping material and the overlying restorative material may be a factor to consider in clinical decision making. However, the clinical relevance of these results should be supported by further studies using natural dentin substrates and long-term aging protocols.

## Figures and Tables

**Table 1:** The materials used in this study and their main chemical compositions, lot numbers, and manufacturers.

Material	Main Chemical Composition	Lot Number	Manufacturer
ProRoot MTA	Tricalcium silicate, dicalcium silicate, tricalcium aluminate, calcium sulfate dihydrate, and bismuth oxide as radiopacifier	0000413824	Dentsply Sirona, Tulsa Dental Specialties, York, PA, USA
Biodentine	Powder: tricalcium silicate, calcium carbonate, zirconium dioxide, calcium oxide, and oxide fillers; liquid: aqueous calcium chloride solution with water-soluble polymer	B34221	Septodont, Saint-Maur-des-Fossés, France
TheraCal LC	Resin-modified calcium silicate material containing Portland cement, strontium glass, fumed silica, barium sulfate, barium zirconate, Bis-GMA, and PEGDMA.	2500002240	Bisco Inc., Schaumburg, IL, USA
MTA BioRep	Tricalcium silicate, dicalcium silicate, tricalcium aluminate, calcium oxide, and calcium tungstate as radiopacifier	75996	Itena Clinical, Paris, France
Fuji II LC	Fluoroaluminosilicate glass, HEMA, polybasic carboxylic acid/polyacrylic acid, urethane dimethacrylate, and dimethacrylate resin components	250201A	GC Corporation, Tokyo, Japan
Equia Forte HT	Powder: strontium fluoroaluminosilicate glass and polyacrylic acid; liquid: aqueous polyacrylic acid	240826A	GC Corporation, Tokyo, Japan
Riva Self Cure	Fluoroaluminosilicate glass, polyacrylic acid, and tartaric acid	1240924	SDI Limited, Bayswater, Victoria, Australia

Note: The compositions are presented as the main components reported in the manufacturer/product documentation. Bis-GMA: bisphenol A-glycidyl methacrylate; PEGDMA: polyethylene glycol dimethacrylate; HEMA: 2-hydroxyethyl methacrylate.

**Table 2:** Descriptive statistics of shear bond strength values according to experimental groups (MPa).

Group	n	Mean	Median	Min	Max	SD
Biodentine–Equia Forte HT	8	3.02	2.95	2.29	4.14	0.62
Biodentine–Riva Self Cure	8	4.26	3.80	2.76	7.35	1.58
Biodentine–Fuji II LC	8	6.67	6.33	5.41	8.67	1.15
MTA BioRep–Riva Self Cure	8	0.39	0.33	0.13	0.96	0.26
MTA BioRep–Equia Forte HT	8	0.37	0.35	0.12	0.71	0.21
MTA BioRep–Fuji II LC	8	1.57	1.64	0.95	2.13	0.46
ProRoot MTA–Equia Forte HT	8	1.78	1.81	1.14	2.29	0.37
ProRoot MTA–Fuji II LC	8	3.97	3.81	3.11	4.82	0.58
ProRoot MTA–Riva Self Cure	8	3.25	3.27	1.94	5.09	1.04
TheraCal LC–Equia Forte HT	8	4.46	4.80	3.12	5.59	0.87
TheraCal LC–Fuji II LC	8	16.41	16.43	12.03	19.83	2.42
TheraCal LC–Riva Self Cure	8	8.99	9.15	7.06	10.48	0.98

Note: n: number of specimens; SD: standard deviation; Min: minimum; Max: maximum; MPa: megapascal.

**Table 3:** Bonferroni-adjusted *p* values for statistically significant pairwise comparisons among experimental groups.

Group 1	Group 2	*p* (Bonf.)
MTA BioRep–Equia Forte HT	ProRoot MTA–Fuji II LC	0.039
MTA BioRep–Equia Forte HT	Biodentine–Riva Self Cure	0.034
MTA BioRep–Equia Forte HT	TheraCal LC–Equia Forte HT	0.012
MTA BioRep–Equia Forte HT	Biodentine–Fuji II LC	<0.001
MTA BioRep–Equia Forte HT	TheraCal LC–Riva Self Cure	<0.001
MTA BioRep–Equia Forte HT	TheraCal LC–Fuji II LC	<0.001
MTA BioRep–Riva Self Cure	ProRoot MTA–Fuji II LC	0.045
MTA BioRep–Riva Self Cure	Biodentine–Riva Self Cure	0.039
MTA BioRep–Riva Self Cure	TheraCal LC–Equia Forte HT	0.014
MTA BioRep–Riva Self Cure	Biodentine–Fuji II LC	<0.001
MTA BioRep–Riva Self Cure	TheraCal LC–Riva Self Cure	<0.001
MTA BioRep–Riva Self Cure	TheraCal LC–Fuji II LC	<0.001
MTA BioRep–Fuji II LC	Biodentine–Fuji II LC	0.012
MTA BioRep–Fuji II LC	TheraCal LC–Riva Self Cure	0.001
MTA BioRep–Fuji II LC	TheraCal LC–Fuji II LC	<0.001
ProRoot MTA–Equia Forte HT	Biodentine–Fuji II LC	0.022
ProRoot MTA–Equia Forte HT	TheraCal LC–Riva Self Cure	0.002
ProRoot MTA–Equia Forte HT	TheraCal LC–Fuji II LC	<0.001
Biodentine–Equia Forte HT	TheraCal LC–Fuji II LC	0.027

Note: *p* (Bonf.): Bonferroni-adjusted *p* value. Pairwise comparisons were performed using Mann–Whitney U test.

**Table 4:** Pairwise comparison results among biomaterials when glass ionomer-based restorative material type was kept constant (Mann–Whitney U test, Bonferroni-adjusted *p* values).

GIC Type	H	*p* (KW)	Pairwise Comparison	*p* (Bonf.)
Fuji II LC	29.096	<0.001	BioRep–ProRoot	0.528
			BioRep–Biodentine	0.004
			BioRep–TheraCal LC	<0.001
			ProRoot–Biodentine	0.528
			ProRoot–TheraCal LC	0.004
			Biodentine–TheraCal LC	0.528
Riva Self Cure	26.518	<0.001	BioRep–ProRoot	0.173
			BioRep–Biodentine	0.019
			BioRep–TheraCal LC	<0.001
			ProRoot–Biodentine	1.000
			ProRoot–TheraCal LC	0.022
			Biodentine–TheraCal LC	0.198
Equia Forte HT	28.161	<0.001	BioRep–ProRoot	0.514
			BioRep–Biodentine	0.002
			BioRep–TheraCal LC	<0.001
			ProRoot–Biodentine	0.420
			ProRoot–TheraCal LC	0.007
			Biodentine–TheraCal LC	0.878

Note: *p* (KW): Kruskal–Wallis test *p* value; *p* (Bonf.): Bonferroni-adjusted *p* value. Pairwise comparisons were performed using Mann–Whitney U test.

**Table 5:** Pairwise comparison results among glass ionomer-based restorative materials when biomaterial type was kept constant (Mann–Whitney U test, Bonferroni-adjusted *p* values).

Biomaterial	H	*p* (KW)	Comparison	*p* (Bonf.)
Biodentine	14.405	<0.001	Equia Forte HT–Riva Self Cure	0.312
			Equia Forte HT–Fuji II LC	<0.001
			Riva Self Cure–Fuji II LC	0.093
MTA BioRep	14.460	<0.001	Equia Forte HT–Riva Self Cure	1.000
			Equia Forte HT–Fuji II LC	0.002
			Riva Self Cure–Fuji II LC	0.004
ProRoot MTA	15.059	<0.001	Equia Forte HT–Riva Self Cure	0.025
			Equia Forte HT–Fuji II LC	<0.001
			Riva Self Cure–Fuji II LC	0.751
TheraCal LC	20.489	<0.001	Equia Forte HT–Riva Self Cure	0.071
			Equia Forte HT–Fuji II LC	<0.001
			Riva Self Cure–Fuji II LC	0.071

Note: *p* (KW): Kruskal–Wallis test *p* value; *p* (Bonf.): Bonferroni-adjusted *p* value. Pairwise comparisons were performed using Mann–Whitney U test.

**Table 6:** Distribution of observed failure modes according to biomaterial–glass ionomer-based restorative material combinations (n = 8).

Biomaterial	GIC	Adhesive	Cohesive (CSC)	Cohesive (GIC)	Mixed	Total
ProRoot MTA	Riva Self Cure	0	8	0	0	8
	Fuji II LC	0	8	0	0	8
	Equia Forte HT	3	5	0	0	8
TheraCal LC	Riva Self Cure	3	0	2	3	8
	Fuji II LC	0	2	3	3	8
	Equia Forte HT	8	0	0	0	8
Biodentine	Equia Forte HT	5	2	0	1	8
	Riva Self Cure	2	4	0	2	8
	Fuji II LC	2	4	0	2	8
MTA BioRep	Equia Forte HT	4	4	0	0	8
	Fuji II LC	2	6	0	0	8
	Riva Self Cure	2	6	0	0	8

Note: CSC: calcium silicate-based biomaterial; GIC: glass ionomer cement.

## Data Availability

The original contributions presented in the study are included in the article, further inquiries can be directed to the corresponding author.
